# The effect of epinephrine on the perfusion index during ultrasound-guided supraclavicular brachial plexus block: a randomized controlled trial

**DOI:** 10.1038/s41598-020-68475-4

**Published:** 2020-07-14

**Authors:** Doyeon Kim, Ji Seon Jeong, Min Jong Park, Justin Sangwook Ko

**Affiliations:** 1 Department of Anesthesiology and Pain Medicine, Samsung Medical Center, Sungkyunkwan University School of Medicine, 81 Irwon-ro, Gangnam-gu, Seoul, 06351 Republic of Korea; 2 Department of Orthopedic Surgery, Samsung Medical Center, Sungkyunkwan University School of Medicine, Seoul, Republic of Korea

**Keywords:** Randomized controlled trials, Physical examination

## Abstract

The perfusion index (PI) is an objective tool used to assess a successful nerve block. Epinephrine is a widely used adjuvant to local anesthetics, and it may affect PI values because of the vasoconstrictive property. The aim of this study was to investigate the influence of epinephrine on PI as an indicator of a successful block in ultrasound-guided supraclavicular brachial plexus block (SCBPB). In this randomized controlled trial, 82 adult patients underwent upper limb surgery under SCBPB were recruited between July 2018 and March 2019 in a single tertiary care center. Participants were randomly assigned to one of two groups: non-epinephrine group (*n* = 41) or epinephrine group (5 mcg ml^−1^, *n* = 41). The primary outcome was the comparison of the “PI ratio 10,” which was defined as the ratio of the PI 10 to the baseline. Receiver operating characteristic (ROC) curves were constructed to determine the accuracy of the PI in predicting the block success at each time interval. The PI ratio 10 was 2.7 (1.9–4.0) in non-epinephrine group and 3.3 (2.2–4.4) in epinephrine group (median difference: 0.4; 95% confidence interval [CI] − 1.1 to 0.2; *P* = 0.207). The ROC curves compared without group identification were not significantly different over time. The cut-off value for the PI and PI ratio at 5 min (PI ratio 5) were 7.7 (area under the ROC [AUROC]: 0.89, 95% CI 0.83–0.94) and 1.6 (AUROC: 0.94, 95% CI 0.90–0.98), respectively. The perineural epinephrine did not affect the PI following a SCBPB. The PI ratio 5 > 1.6 might be considered as a relatively accurate predictor of a successful SCBPB.

Trial registration: This study was registered at the Clinical Trial Registry of Korea (https://cris.nih.go.kr. CriS No. KCT0003006).

## Introduction

The evaluation of peripheral nerve block success is typically assessed using sensory and motor blocks. Recently, several objective methods for the evaluation of block success have been developed to replace conventional methods with inherent limitations (i.e., subjective, time consuming, dependent on patient cooperation, and not applicable under general anesthesia)^1–6^. Among these, the perfusion index (PI) is free of many factors associated with subjective interpretation and can be easily applied to most patients.

The PI is a noninvasive tool for assessing the ratio between pulsatile and non-pulsatile blood flow using a pulse oximeter, and it can also measure sympathetic stimulation or peripheral perfusion with a sensor attached to a finger^7^. It is affected by changes in intravascular volume, elasticity, and intravascular pulse pressure. Therefore, it corresponds to changes in blood volume and varies in values depending on the distensibility of vascular walls and pulse pressure^7^. The blockade of sympathetic nerve fibers after successful nerve block results in increased local blood flow and vasodilation, eventually increasing the PI^8^. Several studies have investigated the utility of the PI for assessing regional anesthesia success and reported that the PI might be used to determine the success of peripheral nerve blocks^2,4,5,9^. However, there is a lack of research to evaluate the usefulness of the PI depending on whether some adjuvants are added to the local anesthetics (LAs). Since the PI is affected by the blood flow in the blood vessels, its value can vary depending on the adjuvants of LAs that affect the condition of blood vessels.

Epinephrine is a widely used adjuvant to LAs to detect unintended anesthetic injections, prolong the duration of sensory blockade in regional anesthesia, and delay the absorption of LAs by inducing vasoconstriction at the injection site^10^. Therefore, epinephrine could lead to vasoconstriction and alter PI values depending on the extent of vasodilation^11^. As a result, the aim of this study was to assess the effect of epinephrine as an adjuvant to local anesthetics on the PI values and also as an indicator of successful supraclavicular brachial plexus block (SCBPB).

## Materials and methods

### Randomization and blinding

A statistician who was unrelated to this study generated a random allocation sequence. The participants were randomly assigned to one of two groups using a computer-generated randomization sequence and a 1:1 ratio with sealed, opaque envelope techniques: a non-epinephrine group and an epinephrine group. Study drugs were mixed with 12.5 ml of 2% lidocaine, 12.5 ml of 0.75% ropivacaine, and 0.1 ml of normal saline in the non-epinephrine group or 12.5 ml of 2% lidocaine, 12.5 ml of 0.75% ropivacaine, and 5 mcg ml^−1^ epinephrine in the epinephrine group. Participants were enrolled by one of the authors (D.K.) and all study drugs were prepared by one of the authors (J.S.J.) who was not involved in either the SCBPB or the outcome assessment. The unilateral upper limb orthopedic surgery was performed by one surgeon (M.J.P.) who was blinded to the allocation groups and clinical patient data were masked until completion of this study.

### Supraclavicular brachial plexus block

On arrival in the block room, standard monitors including electrocardiogram, noninvasive blood pressure (NIBP), heart rate, and pulse oximetry were applied. To avoid influencing any change in blood flow, the NIBP was measured at the ankle; the temperature in the block room was kept constant (25 °C) to minimize the impact from the environment. SCBPB was performed by one expert anesthesiologist with extensive prior experience performing regional anesthesia (J.S.K.). Before the SCBPB, 1.0–1.5 mg of midazolam was administered for anxiolysis. Patients were placed in a semi-fowler’s position with the head turned 45° to the opposite side of the surgical area. Two percent chlorhexidine and 70% isopropyl alcohol were used for skin sterilization. After skin infiltration with 2–3 ml of 1% lidocaine, a high-frequency linear transducer (6–13 MHz; X-porte; SonoSite, Bothell, Washington) was used to identify the brachial plexus in the supraclavicular fossa. A 50-mm, 22-gauge insulated nerve-stimulating needle (PAJUNK^®^, Geisingen, Germany) was inserted following a lateral to medial direction using the in-plane technique. The needle was advanced to the intersection of the first rib and the subclavian artery (i.e., the corner pocket). After confirming that the aspiration test was negative, 10 mg of the study drug were injected. Then the needle was repositioned in the neural cluster; once a threshold current less than 0.5 mA^14^ was confirmed, the remaining 15.1 ml of the study drug were injected.

### Block assessment

After the completion of the SCBPB, the degrees of sensory and motor blocks were assessed every 5 min (min) for a total duration of 30 min. The sensory block was evaluated in the distributions of the radial, median, ulnar, and musculocutaneous nerve dermatomes with the pinprick test using a blunt-tip needle with a three-point scale (2 = normal sensation, 1 = loss of sensation to pinpricks, and 0 = loss of sensation to light touch)^15^. The motor block was tested according to the distribution of nerves as follows: radial (thumb abduction), median (thumb opposition), ulnar (thumb adduction), and musculocutaneous (elbow flexion) with grading on a three-point scale (2 = no loss of force, 1 = decreased force compared with the contralateral arm, and 0 = incapacity to overcome gravity). The block onset time was defined as the time from the completion of the injection of the study drug until a sensory score of 0 was reported, which indicated the complete loss of sensory function in the radial, median, ulnar, and musculocutaneous nerves. Block success was defined as the complete loss of sensory function in the aforementioned nerve distribution within 30 min after completion of the SCBPB.

### Perfusion index assessment

A pulse oximeter (Radical-7^®^; Masimo Corporation, Irvine, CA, USA), a type of attaching tape, was used to monitor the PI. Two separate machines were applied to the middle fingers of each hand to measure the PI (in both the blocked and unblocked arms). The PI was recorded at the following time-points: before the SCBPB (baseline) and every 5 min for 30 min (PI 5, 10, 15, 20, 25, and 30).

The skin temperature was measured on both hands (radial nerve distribution) using a skin thermometer (Hubidic Thermofinder FS 700; ASTERIA Inc., NY, USA). The same surrounding environment and supine positions were maintained until all parameter measurements were completed to prevent changes in the PI.

### Outcomes and statistical analysis

The primary outcome was the comparison of the “PI ratio 10,” which was defined as the ratio of the PI 10 to the baseline. To correct the high skewness of the baseline PI, we used the PI ratio instead of the PI as the primary outcome.

Secondary outcomes were to evaluate the relationship between the complete sensory block and the time of the peak PI, and between the skin temperature of the arm and the PI over time, and to determine the best cutoff value for the PI and PI ratio in the detection of a successful block. The accuracy of the PI to predict the block success was measured at each time interval.

In our preliminary (unpublished) study, the mean ± standard deviation (SD) of the PI ratio 10 was 4.0 ± 1.1 in the epinephrine group (*n* = 5) and 5.5 ± 3 in the non-epinephrine group (*n* = 5). At a power of 0.80 and an alpha error of 0.05, we calculated that 37 patients per group were needed to detect this degree of difference between the two groups. Considering a 10% dropout rate, 82 patients (41 participants for each group) were required to participate in this study.

Continuous variables are presented as the mean ± SDs or as the medians with interquartile ranges (IQRs), as appropriate, and categorical variables are presented as numbers with percentages. The continuous variables were explored for normality using the Shapiro–Wilk test and were compared using the Student’s t-test or the Mann–Whitney U test where appropriate. The categorical variables were compared using a chi-square test or Fisher’s exact test as appropriate. Generalized estimating equations were used to compare the PI between the two groups or between the blocked and unblocked arm with post hoc pairwise comparisons using the Bonferroni test. Because there were no failed blocks, a receiver operating characteristic (ROC) and area under the ROC (AUROC) curve using data of the blocked and unblocked arm was constructed to determine the extent of the PI change and the ability of the PI ratio to predict block success at each time interval. Concurrently, the sensitivity, specificity, positive predictive value, and negative predictive value were calculated. A paired t-test was also used to compare the blocked and unblocked arms at each time point. The relationship between the block onset time and the time of the highest value of the PI, and between the temperature of the blocked arm and the PI in each group were analyzed using Spearman’s correlation. All statistical analyses were performed using the Statistical Package for the Social Sciences (SPSS), version 22.0 (IBM Corp., Chicago, IL). *P-*values were considered statistically significant at the level of *P* < 0.05.

### Ethics

This single-center, prospective, randomized control trial was approved by the Samsung medical center's Institutional Review Board (IRB # SMC 2018-03-060) and written informed consent was obtained from all subjects participating in the trial. The trial was registered prior to patient enrollment at the Clinical Trial Registry of Korea (https://cris.nih.go.kr/cris/, KCT0003006, Principal investigator: Justin Sangwook Ko, Date of registration: July, 20th, 2018). Written informed consent was obtained from all participants before enrollment in this study. We enrolled adult patients between 19 and 76 years of age with American Society of Anesthesiologists Physical Status Classifications from I to III who were scheduled for elective unilateral upper limb orthopedic surgery between July 2018 and March 2019. Exclusion criteria included pregnancy, a body mass index ≥ 30 kg m^−2^, preexisting neurological deficits or neuropathy, those taking α or β blocking agents which can affect vasoconstriction and concentration of epinephrine^12,13^, contraindications to peripheral nerve block, coexisting cardiac and pulmonary problems, diabetes mellitus, and complex regional pain syndrome. All methods were performed in accordance with the relevant guidelines and regulations.

## Results

**Figure 1:**
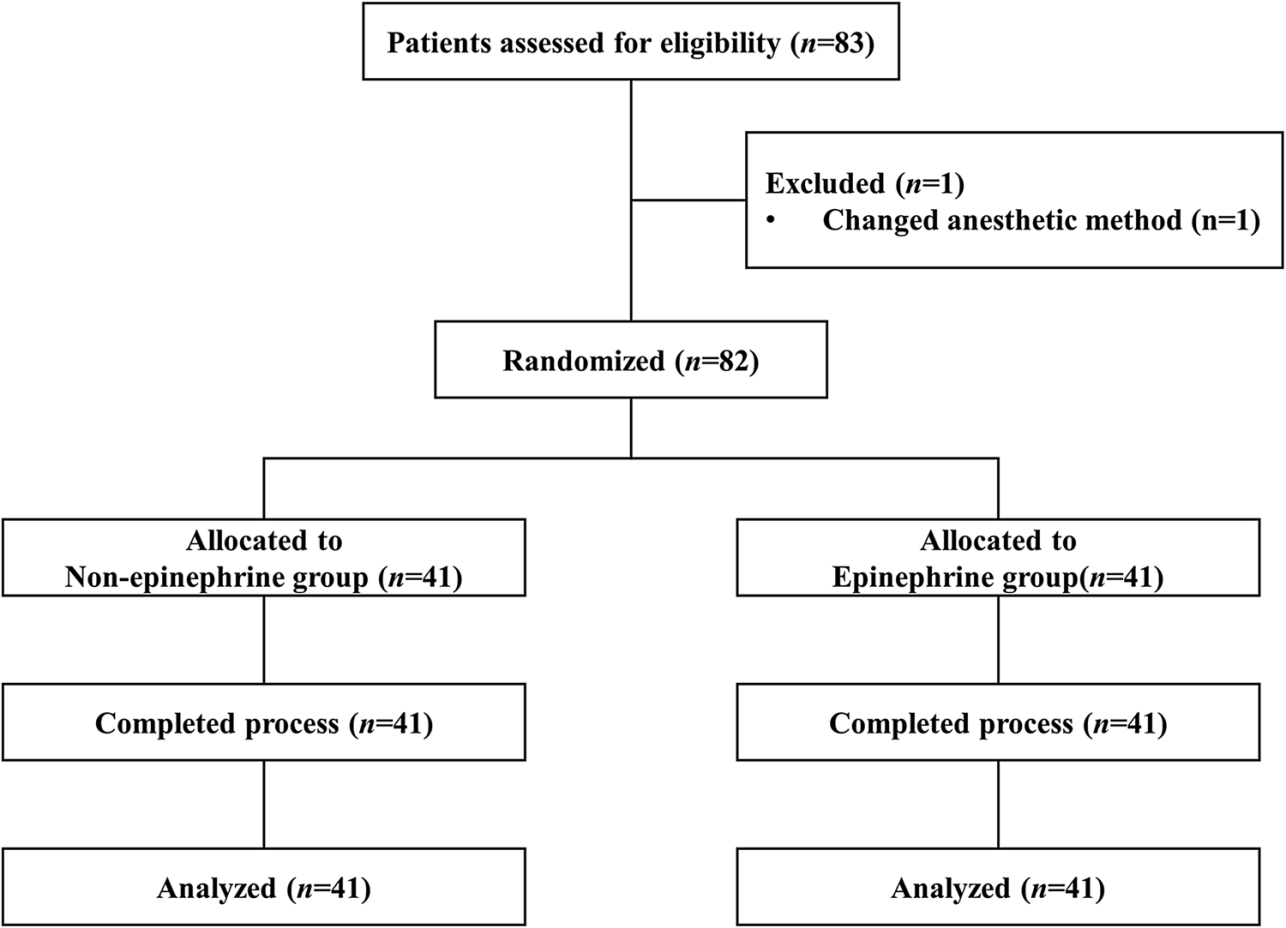
The CONSORT flow diagram.

In total, 83 patients were assessed for eligibility (Fig. 1). Of these, one patient whose anesthetic plan was changed from an SCBPB to monitored anesthesia care was excluded. Thus, 82 patients were finally enrolled and randomly assigned to one of the two groups (*n* = 41, each). There were no differences in the patient characteristics (Table 1) and no failed blocks in these two groups.

**Table 1:** Patient’s characteristics.

	Non-epinephrine (*n* = 41)	Epinephrine (*n* = 41)
Age (years)	45.2 (16.9)	44.8 (17.5)
Female/male, n	19/22	13/28
BMI (kg m^−2^)	24.16 (3.3)	24.4 (2.9)
ASA class (I/II), n	23/18	27/14
**Surgical procedure, n**		
Left/right	13/28	22/19
Wrist	16 (39%)	16 (39%)
Hand	25 (61%)	25 (61%)

Data are expressed as mean (SD) or n (%).

*BMI* body mass index, *ASA* American Society of Anesthesiologists.

**Figure 2:**
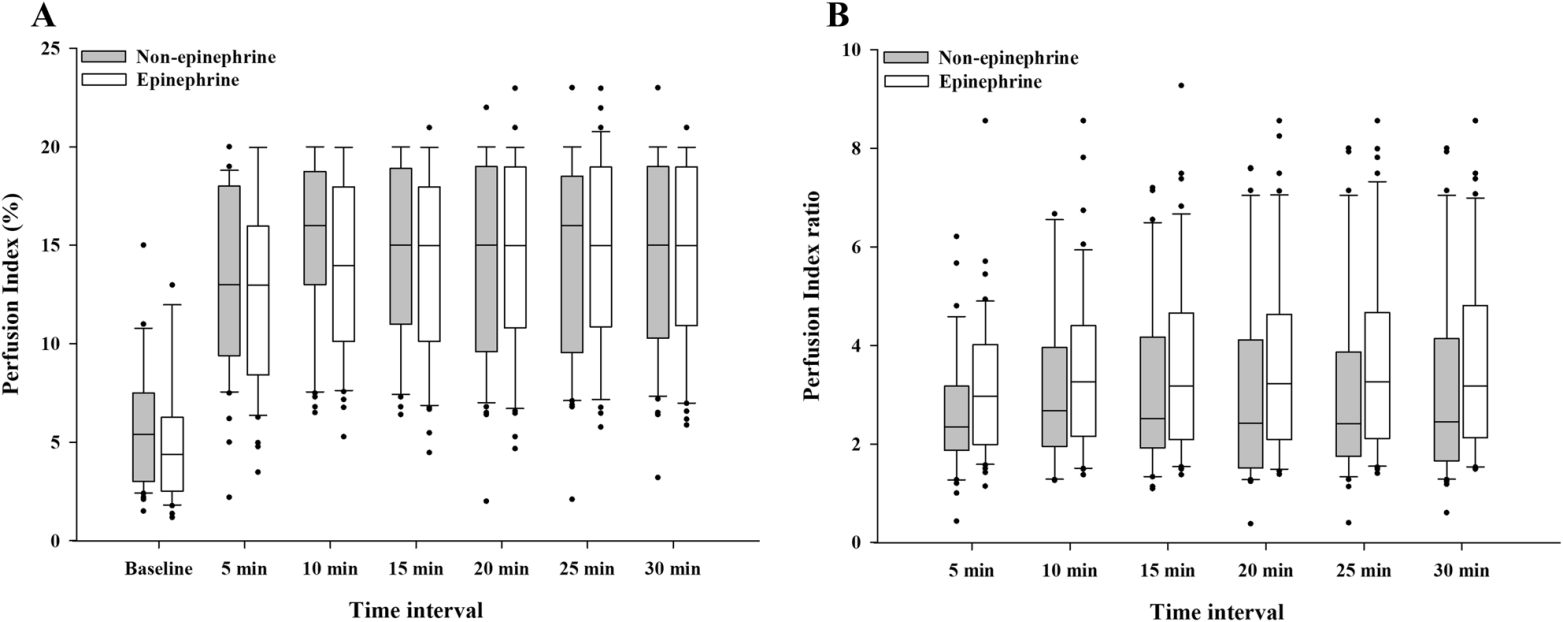
Changes in (**A**) the perfusion index and (**B**) the perfusion index ratio according to the time interval. Boxes represent the medians with the 25th/75th percentiles. Whiskers represent the minimum/maximum values, excluding outliers. Points represent the outliers.

There were no differences in the PI and PI ratios between the two groups according to the time interval (*P* = 0.894 and *P* = 0.079, respectively) (Fig. 2). The PI values increased in both groups after 5 min compared with the baseline (both groups, *P* < 0.001). The median (IQR) of the PI ratio 10 was 2.7 (1.9–4.0) in the non-epinephrine group and 3.3 (2.2–4.4) in the epinephrine group (median difference: 0.4; 95% confidence interval [CI] − 1.1 to 0.2; *P* = 0.207).

**Figure 3:**
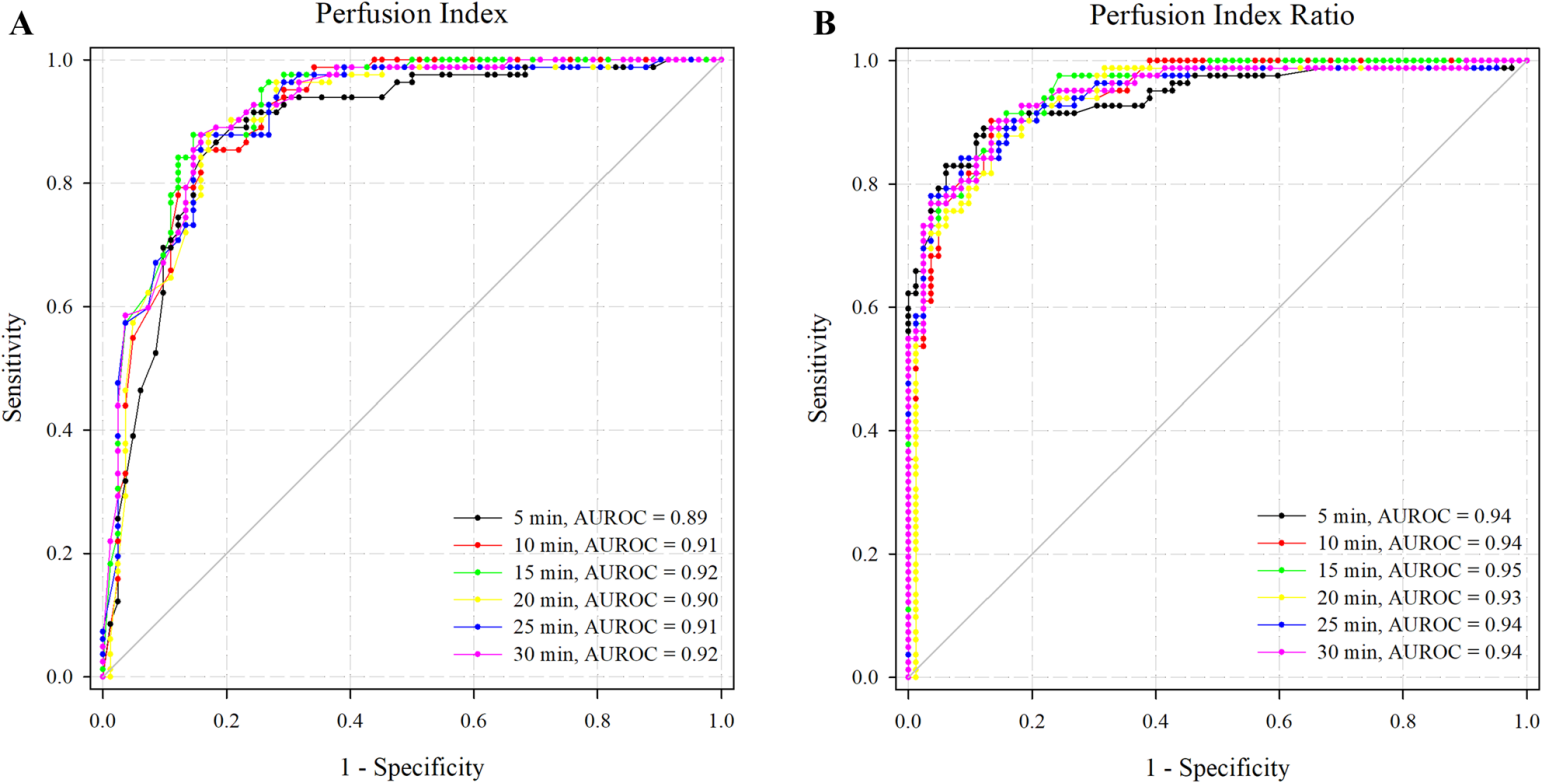
The ROC curve of the PI and PI ratio over time intervals without group identification. *AUROC* area under the receiver operating characteristic curve.

Because the PI and PI ratios were not significantly different between the two groups, the ROC curves were compared without group identification, and they showed no significant differences over time (Fig. 3). Subsequently, both the PI and PI ratio at 5 min (PI ratio 5) demonstrated a good ability to predict block success. The AUROC for the PI and PI ratio 5 were 0.89 (95% CI 0.83–0.94) with a cut-off value > 7.7 and 0.94 (95% CI 0.90–0.98) with a cut-off value > 1.6, respectively. Sensitivity and specificity were as follows: 0.89 (95% CI 0.83–0.94) and 0.79 (95% CI 0.69–0.87) in PI at 5 min, 0.87 (95% CI 0.79–0.94) and 0.89 (95% CI 0.80–0.95) in PI ratio 5, respectively.

There was no significant correlation between the time to reach the highest PI value and the time of block onset (non-epinephrine group: correlation coefficient = − 0.011, *P* = 0.947; epinephrine group: correlation coefficient = 0.177, *P* = 0.269, respectively). In addition, the skin temperature and PI were not significantly correlated (non-epinephrine group: correlation coefficient = 0.201, *P* = 0.206; epinephrine group: correlation coefficient = − 0.115, *P* = 0.476, respectively). The times to reach peak PI, block onset, and peak skin temperature were not statistically difference between the two groups (*P* = 0.341*, P* = 0.851*,* and* P* = 0.730*,* respectively). Regardless of the group, the times to reach peak PI, block onset, and peak skin temperature were 13.8 ± 7.7 min, 14.6 ± 5.8 min, and 16.7 ± 7.9 min, respectively.

A subgroup analysis revealed that the changes in the PI and PI ratio between blocked and unblocked arms were statistically different over time in both groups (*P* < 0.001) (Table 2). No participants experienced complications or adverse effects related to the SCBPB.

**Table 2:** Perfusion index and perfusion index ratio of blocked and unblocked arm in each group.

	Non-epinephrine (*n* = 41)			Epinephrine (*n* = 41)		
	Blocked arm	Unblocked arm	*P* value	Blocked arm	Unblocked arm	*P* value
**Perfusion index (%)**						
Baseline	5.4 (3.0–7.5)	5.0 (3.0–6.5)	0.532	4.4 (2.6–6.3)	3.7 (2.5–6.5)	0.822
5 min	13.0 (9.4–18.0)	4.9 (3.0–6.1)	< 0.001	13.0 (8.5–16.0)	4.7 (3.2–7.3)	< 0.001
10 min	16.0 (13.0–18.8)	5.0 (3.2–7.8)	< 0.001	14.0 (10.2–18.0)	4.4 (3.1–7.9)	< 0.001
15 min	15.0 (11.0–18.9)	4.2 (2.5–6.6)	< 0.001	15.0 (10.2–18.0)	4.6 (3.1–7.6)	< 0.001
20 min	15.0 (9.6–19.0)	4.4 (2.5–6.0)	< 0.001	15.0 (10.9–19.0)	4.3 (3.0–7.3)	< 0.001
25 min	16.0 (9.6–18.5)	4.4 (2.5–7.4)	< 0.001	15.0 (10.9–19.0)	4.8 (3.1–7.6)	< 0.001
30 min	15.0 (10.3–19.0)	4.2 (2.6–6.8)	< 0.001	15.0 (11.0–19.0)	4.6 (3.3–7.6)	< 0.001
**Perfusion index ratio**						
5 min	2.3 (1.9–3.2)	1.0 (0.8–1.4)	< 0.001	3.0 (2.0–4.0)	1.2 (1.0–1.4)	< 0.001
10 min	2.7 (1.9–4.0)	1.1 (0.9–1.4)	< 0.001	3.3 (2.2–4.4)	1.2 (1.0–1.4)	< 0.001
15 min	2.5 (1.9–4.2)	0.98 (0.7–1.4)	< 0.001	3.2 (2.1–4.7)	1.2 (1.0–1.3)	< 0.001
20 min	2.4 (1.5–4.1)	1.0 (0.7–1.3)	< 0.001	3.2 (2.1–4.6)	1.1 (1.0–1.3)	< 0.001
25 min	2.4 (1.8–3.9)	1.0 (0.7–1.4)	< 0.001	3.3 (2.1–4.7)	1.2 (1.0–1.4)	< 0.001
30 min	2.5 (1.7–4.1)	1.03 (0.7–1.3)	< 0.001	3.2 (2.1–4.8)	1.2 (1.0–1.3)	< 0.001

Data are expressed as median (IQR).

## Discussion

In this study, perineurally injected epinephrine combined with ropivacaine did not have any influence on either the PI or the PI ratio after the SCBPB in patients undergoing forearm surgeries. However, the PI and PI ratio 5 showed a good ability to predict a successful SCBPB. There was no significant correlation between the times to reach peak PI and complete sensory block, or between the skin temperature and the PI values of the blocked arm.

Previous studies have shown that the PI is a useful indicator to predict successful peripheral nerve blocks^2,4,5^. However, it is speculated that the use of adjuvants with local anesthetics can change the degree of vasodilation and theoretically affect the PI values. Epinephrine is one of the most popular adjuvants in peripheral nerve blocks and is known to produce vasoconstriction and subsequently alter PI values. The peripheral nervous system receives dual blood supply from blood vessels in the endoneurium and also in the epineurial space^16^. Of these, the extrinsic blood supply in response to adrenergic stimulation is the action site of epinephrine^16^ and it results in a marked decrease in blood flow^17^. However, in our study, the PI and PI ratio showed no difference over time regardless of the administration of epinephrine. Perineurally injected epinephrine is slowly absorbed to alter heart rate and blood pressure, but has no effect on the mean arterial pressure^17^. Since the mean arterial pressure is important when assessing the relationship between systemic resistance, blood flow, and pressure, this might be one of the reasons for the unaffected PI and PI ratio when epinephrine is injected perineurally.

Because of the high variability of the PI values, the PI ratio might be more accurate than the absolute value of PI^4,18^. For example, Abdelnasser et al*.* determined the PI ratio 10 as an indicator of a successful block, and suggested that the cut-off values of the PI and PI ratio 10 were 3.3 and 1.4, respectively^4^. However, in our study, the PI ratio 5 was determined as an indicator of a successful block, and the cut-off values for the PI and PI ratio 5 were 7.7 and 1.6, respectively. A previous study measured the PI values at relatively wide intervals (10 min), but we used shorter intervals (5 min). Although there were no mentions of the PI ratio, previous studies have compared the baseline PI with the PI value after 5 min and reported its statistical significance^19–21^. In addition, it was confirmed that the PI at 5 min after the SCBPB was the fastest reference point for the change in the PI as a result of AUROC analysis, and the AUROCs were not significantly different across time intervals. Therefore, we recommend the PI ratio 5 as an indicator of successful blocks. PI values may vary depending on the instrument used to measure the PI. However, the PI ratio can be expressed as a constant value because it shows the ratio of the baseline value and each time interval despite the aforementioned difference. In practice, although the cuff-off value of PI was more than two times higher, the cut-off value of the PI ratio was similar to that found in a previous study. Therefore, a PI ratio 5 > 1.6 in our study can be useful predictors of the block success of an SCBPB. In addition, future study is warranted to approve the predictive value of PI ratio 5.

Previous methods used to predict block success included skin temperature^8^, laser doppler perfusion imager^22^, skin electrical resistance^23^, and the PI^4^. The skin temperature and PI can be measured relatively easily compared to other methods. In our study, the time to reach the highest PI (14 min) was not correlated with the time to reach the highest skin temperature (17 min). Although the assessment of skin temperature is regarded as a reliable and objective method to evaluate block success, the time required to reach the peak value was also longer than that of the PI. For example, in epidural-induced sympathectomy, the PI was shown to be a faster and more sensitive indicator of block success than the skin temperature^8^. This outcome may be due to a discrepancy in skin blood flow control during the changes in peripheral vasodilation^24^.

There are several limitations to this study. First, for the ROC curve analysis, data from both the blocked and unblocked arm of the same patient were used. In previous studies, data from successful and failed blocks were used. However, in our study, all SCBPBs were successful, so the data of the unblocked arms were regarded as the failed block data. Second, in the current study, we could not suggest the validated PI as an objective indicator of the successful regional anesthesia. However, the PI ratio 5 was demonstrated as a surrogate endpoint and may be a useful indicator in clinical situations. Third, we administered midazolam to ease patient’s anxiety before the SCBPB. Midazolam, a short-acting benzodiazepine, can lead to peripheral vasodilation^25^ and produce dose-dependent relaxation^26^, and may increase PI and overestimate the value. Lastly, we added 5 mcg ml^−1^ of epinephrine to the ropivacaine. Although this dosage is frequently used in clinical practice, it is expected that its extent of influence on the degree of vasoconstriction might vary with the varying dose of epinephrine. Future research is needed to determine the dose of epinephrine that can be used to maintain the reliability of the PI.

In conclusion, the use of epinephrine as an adjuvant to local anesthetics did not affect the PI and PI ratio. The PI ratio is a useful indicator for evaluating a successful SCBPB regardless of the presence of epinephrine. A PI ratio at 5 min > 1.6 is suggested as a relatively accurate predictor of a successful SCBPB.
